# FFAR4 activation inhibits lung adenocarcinoma via blocking respiratory chain complex assembly associated mitochondrial metabolism

**DOI:** 10.1186/s11658-024-00535-3

**Published:** 2024-01-19

**Authors:** Zhe Wang, Jinyou Li, LongFei Wang, Yaowei Liu, Wei Wang, JiaYao Chen, HuiJun Liang, Y. Q. Chen, ShengLong Zhu

**Affiliations:** 1 Wuxi School of Medicine, Jiangnan University, Wuxi, China; 2 Department of Thoracic Surgery, Affiliated Hospital of Jiangnan University, Wuxi, China; 3 The First Affiliated Hospital of Ningbo University, Ningbo, China; 4 State Key Lab of Food Science and Resources, Jiangnan University, Wuxi, China

**Keywords:** FFAR4, LUAD, OXPHOS, Metabolism reprogramming

## Abstract

Despite notable advancements in the investigation and management of lung adenocarcinoma (LUAD), the mortality rate for individuals afflicted with LUAD remains elevated, and attaining an accurate prognosis is challenging. LUAD exhibits intricate genetic and environmental components, and it is plausible that free fatty acid receptors (FFARs) may bridge the genetic and dietary aspects. The objective of this study is to ascertain whether a correlation exists between FFAR4, which functions as the primary receptor for dietary fatty acids, and various characteristics of LUAD, while also delving into the potential underlying mechanism. The findings of this study indicate a decrease in *FFAR4* expression in LUAD, with a positive correlation (*P* < 0.01) between FFAR4 levels and overall patient survival (OS). Receiver operating characteristic (ROC) curve analysis demonstrated a significant diagnostic value [area under the curve (AUC) of 0.933] associated with *FFAR4* expression. Functional investigations revealed that the FFAR4-specific agonist (TUG891) effectively suppressed cell proliferation and induced cell cycle arrest. Furthermore, FFAR4 activation resulted in significant metabolic shifts, including a decrease in oxygen consumption rate (OCR) and an increase in extracellular acidification rate (ECAR) in A549 cells. In detail, the activation of FFAR4 has been observed to impact the assembly process of the mitochondrial respiratory chain complex and the malate–aspartate shuttle process, resulting in a decrease in the transition of NAD^+^ to NADH and the inhibition of LUAD. These discoveries reveal a previously unrecognized function of FFAR4 in the negative regulation of mitochondrial metabolism and the inhibition of LUAD, indicating its potential as a promising therapeutic target for the treatment and diagnosis of LUAD.

**Supplementary Information:**

The online version contains supplementary material available at 10.1186/s11658-024-00535-3.

## Introduction

Lung cancer ranks first among all malignant tumors in terms of both morbidity and mortality [1–3]. Adenocarcinoma of the lung is the prevailing histologic subtype observed in cases of lung cancer [4, 5]. Despite notable advancements in the study and management of lung adenocarcinoma (LUAD), the mortality rate for individuals afflicted with LUAD remains elevated, and the attainment of an accurate prognosis is challenging [6–9]. Consequently, there is an urgent requirement for a novel prognostic biomarker for LUAD, as well as targeted therapies exhibiting heightened sensitivities and specificities.

The suboptimal survival rate and elevated mortality associated with LUAD may be attributed to a multifaceted interplay between genetic factors and environmental influences [10]. G protein-couple receptors (GPCRs) have emerged as highly promising targets for drug development and have been extensively investigated [11–13]. A growing body of research indicates that several GPCRs are implicated in the development and progression of various cancer types, and they exhibit a dual role in cancer and metabolism [14–16]. Notably, the free fatty acid receptors FFAR1 (GPR40), FFAR2 (GPR43), FFAR3 (GPR41), and FFAR4 (GPR120) have the potential to bridge the genetic and environmental factors associated with cancer [17–20]. Activation of FFAR2 and FFAR3 occurs through short-chain fatty acids, while FFAR1 and FFAR4 are activated by medium-chain and long-chain fatty acids [21, 22]. Fatty acids, particularly long-chain fatty acids (LCFAs), are major constituents of the human diet [23, 24].

Several studies have demonstrated that a LCFA-rich diet is associated with increased survival rates and enhanced immune responses in LUAD [25–27]. FFAR4, which is highly abundant in the intestines and lungs, primarily functions as the receptor for LCFAs. However, the majority of FFAR4 research has focused on its role in the digestive tract [28–30], neglecting its potential contributions to the lungs, where it is also highly expressed. Consequently, the involvement of FFAR4 in lung adenocarcinoma remains uncertain.

In the current investigation, it was observed that the expression of *FFAR4* was notably diminished in individuals diagnosed with lung adenocarcinoma, and this decrease in FFAR4 levels was found to be correlated with a more unfavorable prognosis. Additionally, the activation of FFAR4 was found to significantly impede the proliferation of lung adenocarcinoma cells, and this effect was mediated through its influence on mitochondrial metabolism.

## Materials and methods

### Datasets

We obtained clinical data and RNA sequence results from a total of 535 cases of lung adenocarcinoma from The Cancer Genome Atlas (TCGA) database, along with 59 corresponding samples of paracancerous tissue. The data obtained were subsequently transformed into the transcripts per million (TPM) format for further analysis. Additionally, TPM-formatted RNA-sequencing (RNA-seq) data from TCGA and Genotype—Tissue Expression (GTEx) databases were downloaded from UCSC Xena. Furthermore, the GSE118370 and GSE40275 GEO datasets were examined to assess the expression of *FFAR4* mRNA. GEPIA was used to evaluate FFAR4 expression in different cancers [31]. Data on FFAR4 expression levels in different cell lines from ATCC were obtained from the Cancer Cell Line Encyclopedia (CCLE) database.

### Human tissue specimens

This research was granted approval by the Ethics Committee of The First Affiliated Hospital of Ningbo University, and written informed consent was obtained from all participants involved in the study. The tissues were obtained from the First Affiliated Hospital of Ningbo University (no. NBU2022-078A). Frozen tumor samples were utilized to extract total RNA using the TRIzol method. The confirmation of all tumor tissues was conducted by two experienced pathologists. The clinicopathological characteristics of the patients with LUAD are presented in Table 1.

**Table 1:** Correlation between FFAR4 expression and clinicopathological characteristics in LUAD.

Characteristic	Low expression of FFAR4	High expression of FFAR4	*p*
*n*	267	268	
T stage, *n* (%)			0.127
T1	77 (14.5%)	98 (18.4%)	
T2	151 (28.4%)	138 (25.9%)	
T3	25 (4.7%)	24 (4.5%)	
T4	13 (2.4%)	6 (1.1%)	
N stage, *n* (%)			0.109
N0	168 (32.4%)	180 (34.7%)	
N1	48 (9.2%)	47 (9.1%)	
N2	45 (8.7%)	29 (5.6%)	
N3	0 (0%)	2 (0.4%)	
M stage, *n* (%)			0.343
M0	188 (48.7%)	173 (44.8%)	
M1	16 (4.1%)	9 (2.3%)	
Pathologic stage, *n* (%)			0.093
Stage I	140 (26.6%)	154 (29.2%)	
Stage II	55 (10.4%)	68 (12.9%)	
Stage III	50 (9.5%)	34 (6.5%)	
Stage IV	16 (3%)	10 (1.9%)	
Primary therapy outcome, *n* (%)			0.051
PD	43 (9.6%)	28 (6.3%)	
SD	22 (4.9%)	15 (3.4%)	
PR	2 (0.4%)	4 (0.9%)	
CR	151 (33.9%)	181 (40.6%)	
Gender, *n* (%)			0.022
Female	129 (24.1%)	157 (29.3%)	
Male	138 (25.8%)	111 (20.7%)	
Race, *n* (%)			0.431
Asian	4 (0.9%)	3 (0.6%)	
Black or African American	32 (6.8%)	23 (4.9%)	
White	201 (42.9%)	205 (43.8%)	
Age, *n* (%)			0.597
≤ 65	131 (25.4%)	124 (24%)	
>65	127 (24.6%)	134 (26%)	
Residual tumor, *n* (%)			0.611
R0	181 (48.7%)	174 (46.8%)	
R1	7 (1.9%)	6 (1.6%)	
R2	1 (0.3%)	3 (0.8%)	
Anatomic neoplasm subdivision, *n* (%)			0.296
Left	95 (18.3%)	110 (21.2%)	
Right	162 (31.2%)	153 (29.4%)	
Anatomic neoplasm subdivision2, *n* (%)			0.833
Central lung	33 (17.5%)	29 (15.3%)	
Peripheral lung	64 (33.9%)	63 (33.3%)	
Number_pack_years_smoked, *n* (%)			0.066
<40	89 (24.1%)	99 (26.8%)	
≥ 40	104 (28.2%)	77 (20.9%)	
Smoker, *n* (%)			< 0.001
No	22 (4.2%)	53 (10.2%)	
Yes	238 (45.7%)	208 (39.9%)	
Age, median (IQR)	65 (59.25, 72)	67 (59, 73)	0.328

*PD* Progressive Disease,* SD* Stable Disease,* PR* Partial Response,* CR* Complete Response

### Cell culture and treatments

The human bronchial epithelial cells (BEAS-2B) and human LUAD cell lines (A549 & H1299) were procured from the National Collection of Authenticated Cell Cultures and cultured in Dulbecco’s modified Eagle medium (DMEM; Gibco, C11965500) supplemented with 5% fetal bovine serum (FBS; VivaCell, Shanghai, C04001, China). The cells were maintained at a temperature of 37 °C in an incubator with a 5% CO_2_ flush. Different concentrations of TUG891, dissolved in DMSO, were administered to the cells, while the vehicle incubations received an equivalent volume of DMSO. The transfection procedure was carried out when the cells grew to a confluence degree of 60–70% using the jetPRIME transfection reagent (Polyplus transfection) according to the manufacturer’s instructions. The transfection groups were as follows: the vehicle group (pcDNA3.1) and treatment group (pcDNA3.1-FFAR4). In our experiments, three-well repetitions were set in each group in every experiment. All experiments were conducted in triplicate.

### In vitro cell proliferation and cell cycle assays

The CCK8 assay was employed to ascertain the cellular proliferation. The cells were introduced into 96-well plates and subsequently exposed to either the transfection plasmid or TUG891, followed by an incubation period of 72 h. Subsequently, 20 μl of a 5 mg/ml CCK8 solution was added and incubated for an additional hour. The optical density (OD) was measured at 450 nm. Flow cytometry was utilized to analyze the distribution of cell cycle phases. Specifically, cells were subjected to treatment with or without 1 μM L7G for a duration of 24 h, after which they were trypsinized, washed with PBS, and fixed in cold 80% ethanol. Following the elimination of ethanol through centrifugation, the cells were subjected to staining with PI/RNase Staining Buffer (BD Biosciences, USA) for a duration of 20 min in a light-restricted environment, adhering to the guidelines provided by the manufacturer. The quantification of DNA content was conducted using a flow cytometer manufactured by Beckman Coulter.

### Oxygen consumption rate (OCR) and extracellular acidification rate (ECAR) analysis

For the measurement of OCR, the cells were treated with 500 µL of assay medium consisting of XF basal medium (Seahorse, 102353), 1 mM sodium pyruvate, 1 mM l-glutamine, and 10 mM glucose. Port injection was carried out using 1 mM oligomycin, 3 mM SCCP, 0.5 mM antimycin, and rotenone. On the other hand, for ECAR measurements, the cells were treated with 500 µL of assay medium consisting of XF basal medium and 1 mM l-glutamine. Port injection was performed using 10 mM glucose, 1 mM oligomycin, and 50 mM 2-deoxy-d-glucose.

### Quantitative real-time polymerase chain reaction (qPCR)

Following the manufacturer’s protocol, total RNA was isolated using RNAiso Plus (Takara), and then reverse-transcribed using Prime Script™ RT Master Mix (Takara). The mRNA quantification was conducted utilizing the CFX Connect™ Real-Time PCR Detection System (Bio-Rad) and Hieff UNICON qPCR SYBR green master mix (Yeasen Biotechnology, 11198ES). The qPCR protocol consisted of 40 cycles of denaturation at 95 °C for 15 s, followed by annealing and extension at 60 °C for 30 s. The obtained results were normalized to the housekeeping gene β-actin and expressed as 2^−ΔΔCt^.

### Transcriptomics analysis

Total RNA extraction from A549 cells was carried out using a K101 total RNA Extraction kit (JN. BIOTOOLS). Reverse transcription was performed using a K102 BT-I 1st Strand cDNA Synthesis kit (JN. BIOTOOLS), and second-strand synthesis was accomplished using a Second Strand cDNA Synthesis kit (Beyotime Biotechnology, D7172). DNA samples were fragmented and tagged with Tn5 transposase. Subsequently, polymerase chain reaction (PCR) amplification was conducted using HiFi PCR Mix for NGS (CWBIO and CW2648), with index codes being assigned to each sample. The resulting PCR products were purified using a FastPure Gel DNA Extraction Mini kit (Nanjing Vazyme Biotech Co, Ltd, DC301-01) and subjected to sequencing using Illumina NovaSeq (GENEWIZ). The obtained sequencing data were subjected to analysis using STAR (http://code.google.com/p/rna-star/) and R software (version 3.5). Genes exhibiting differential expression were defined as those with a *P* value < 0.05 and a fold change ≥ 2.

### Intracellular NAD^+^/NADH measurement

The concentration of NADH and NAD^+^ was determined using the NAD^+^/NADH Assay Kit with WST-8 (Beyotime Biotechnology). Specifically, A549 cells were cultured in six-well plates at a density of 1.0 × 10^6^ cells per well and incubated overnight. Subsequently, the culture medium was replaced with fresh medium containing FIGs, FIGs-L, or FIGs-LC at a concentration of 0.3 mg/ml. After a 4-h incubation period, the cells were washed and 200 μL of an extracting solution was added. Finally, the concentration of NADH or NAD^+^ was measured using a multiplate reader.

### LinkedOmics analysis

The LinkedOmics database, accessible at (http://www.linkedomics.org/login.php), is a web-based platform that offers a comprehensive tool for multiomics data analysis. It directly connects to TCGA database[32]. In this study, RNA-seq data from a total of 515 lung adenocarcinoma patients were obtained from TCGA and analyzed using the LinkedOmics website. This analysis resulted in the identification of a coexpressed gene list for *FFAR4*. Subsequent data analysis of the *FFAR4* coexpressed genes, including Gene Ontology (GO) analysis and Gene Set Enrichment Analysis (GSEA), was also conducted using the LinkedOmics platform. Pearson correlation coefficients were employed, and a rank criterion of FDR < 0.05 was applied.

### Nontargeted metabolomics analysis

Nontargeted metabolomics on cell lysates was accomplished by the Wuxi School of Medicine at Jiangnan University of China. The analysis of cell metabolites followed a previously described protocol. Briefly, A549 cell samples (50 mg) were subjected to compound extraction using a mixture of methanol/acetonitrile/deionized water (2:1:1, v/v/v). The resulting mixture was then centrifuged at 12,000*g*, 4 °C for 20 min. The supernatant (500 μl) was concentrated using a vacuum centrifugal concentration dryer for 4 h and resuspended in 150 μl methanol/deionized water (4:1, v/v) prior to analysis. Instrumental analysis was performed using an ultra-performance liquid chromatography and Q-Exactive high-resolution mass spectrometer system (Thermo Fisher Scientific, Waltham, MA) with a C18 column (Waters Corporation, Milford, MA). The Compound Discover 3.2 software, developed by Thermo Fisher Scientific (Waltham, MA) was employed for the purposes of metabolite extraction, quantification, and relative abundance analysis of the raw measurement data. To discern the dissimilarities between the vehicle and FFAR4 activation groups, the principal component analysis (PCA) and orthogonal partial least-squares discriminant analysis (OPLS-DA) models were employed. Metabolic features exhibiting variable importance in projection (VIP > 1 and p < 0.05) were deemed potential differential biomarkers. Metabolic pathway analysis based on the identified metabolites was performed using MetaboAnalyst 5.0 (https://www.metaboanalyst.ca/; accessed on 26 September 2022).

### Statistical analysis

Statistical analyses were conducted using R version 3.6.3. The R ClusterProfiler package was utilized to analyze the GO function and KEGG pathway enrichment, while the ggplot2 package was employed for data visualization. To evaluate the relationship between *FFAR4* expression and the clinicopathological characteristics of patients, Fisher’s exact test, Wilcoxon rank sum test (WRST), chi-square test, and logistic regression analyses were performed. Additionally, Kaplan–Meier analysis was conducted on TCGA database to investigate the potential association between *FFAR4* expression and overall survival (OS). The clinical and genetic associations between the two were evaluated through univariate and multivariate analyses, while patient survival was assessed using Cox proportional hazards models. The data were presented as the mean ± standard error of the mean (SEM) of three independent repetitions. All cell-based data were analyzed using a two-tailed Student’s *t*-test with SPSS (SPSS Inc.). A significance level of *P* < 0.05 denoted statistically significant disparities.

## Results

### *FFAR4* expression is significantly decreased in LUAD

**Fig. 1:**
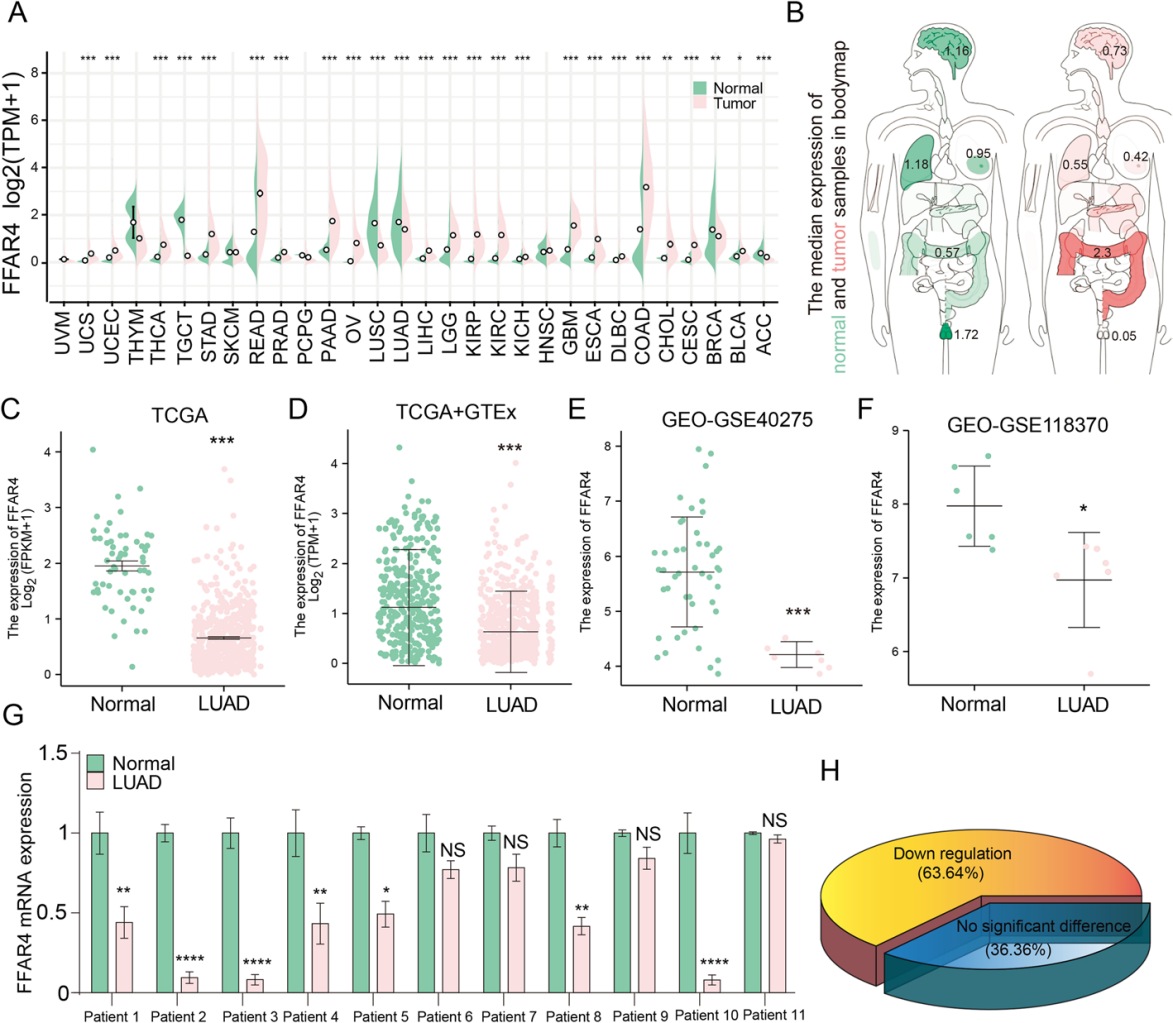
*FFAR4* expression is significantly decreased in LUAD. **A** *FFAR4* levels in different tumors were assessed using TCGA database. **B** The median expression of *FFAR4* between normal and tumor samples in bodymap from the GEPIA. **C** Tumor tissue and control tissue samples in TCGA database were compared for *FFAR4* expression. **D** A comparison of *FFAR4* expression between normal tissue samples from GTEx and control and normal tissue samples from TCGA database was performed using WRST. **E**–**F** Analysis of GEO-GSE40275 and GEO-GSE118370 datasets confirms the presence of *FFAR4* mRNA deregulation in LUAD. **G**–**H** Expression of *FFAR4* mRNA in lung adenocarcinoma and paracarcinoma tissues of 11 patients with LUAD from the First Affiliated Hospital of Ningbo University. Data are expressed as the mean ± SEM. *P* < 0.05 was considered statistically significant using two-way analysis of variance (ANOVA); NS, not significant

Pancancer analysis revealed that the *FFAR4* were differentially expressed in tumor and paracarcinoma tissues. Among the 30 distinct types of tumors examined, *FFAR4* was upregulated in five tumors and downregulated in 20 tumors, including LUSC and LUAD (all *P* < 0.05; Fig. 1A**)**. Data from the GEPIA database showed that *FFAR4* was most widely distributed in five tissues, in the order of testis > lung > brain > breast > colorectal in normal tissues. Notably, all five tumor tissues displayed aberrant FFAR4 expression levels in comparison with their corresponding normal tissues (Fig. 1B). Lung cancer (LC) and colorectal cancer (CRC) were found to be prominently ranked among the most lethal cancers in both males and females among the five cancer types investigated [33]. The literature has extensively examined the involvement of FFAR4 in colorectal cancer [34], while its association with lung cancer remains insufficiently elucidated. Lung cancer is commonly categorized into small cell lung cancer (SCLC) and non-small cell lung cancer (NSCLC), with the latter comprising a substantial proportion of all lung cancer cases, primarily characterized by lung adenocarcinoma (LUAD) [35].

Utilizing data from TCGA database, a notable downregulation of *FFAR4* expression was observed in 535 LUAD tissue samples in comparison with 59 normal control tissue samples (*P* < 0.001; Fig. 1C). The GTEx and TCGA database and GEO database also provided similar results in LUAD (Fig. 1D–F). Subsequently, the expression of *FFAR4* mRNA was validated in both lung adenocarcinoma and paracancerous tissue obtained from patients with LUAD. Consistent with expectations, a decrease in FFAR4 expression was observed in 63.64% of patients (Fig. 1G, H). Furthermore, a correlation between *FFAR4* expression and various clinicopathological factors (Additional file 1: Fig. S1A-G, Table 2) was identified, suggesting a potential association between abnormal FFAR4 expression and the progression of LUAD.

**Table 2:** The expression of FFAR4 has been Linked with the clinicopathological features (Logistic Regression)

Characteristics	Total (*N*)	Odds ratio (OR)	*P* value
T stage (T2, T3, and T4 versus T1)	532	0.774 (0.538–1.112)	0.167
N stage (N1, N2, and N3 versus N0)	519	0.900 (0.624–1.299)	0.575
M stage (M1 versus M0)	386	0.591 (0.245–1.347)	0.221
Pathologic stage (stage III and stage IV versus stage I and stage II)	531	0.674 (0.440–1.028)	0.049
Primary therapy outcome (PR and CR versus PD and SD)	446	1.923 (1.240-3.008)	**0.004**
Gender (male versus female)	535	0.681 (0.484–0.958)	**0.028**
Race (white versus Asian and Black or African American)	468	1.385 (0.809–2.397)	0.238
Age (>65 versus ≤ 65)	516	1.168 (0.827–1.651)	0.378
Residual tumor (R1 and R2 versus R0)	372	1.184 (0.443–3.219)	0.735
Anatomic neoplasm subdivision (right versus left)	520	0.765 (0.537–1.087)	0.136
Anatomic neoplasm subdivision2 (peripheral lung versus central lung)	189	1.120 (0.610–2.065)	0.715
number_pack_years_smoked (≥ 40 versus <40)	369	0.555 (0.366–0.838)	**0.005**
Smoker (yes versus no)	521	0.266 (0.148–0.458)	**<0.001**

### Higher *FFAR4* expression informed better patient survival

**Fig. 2:**
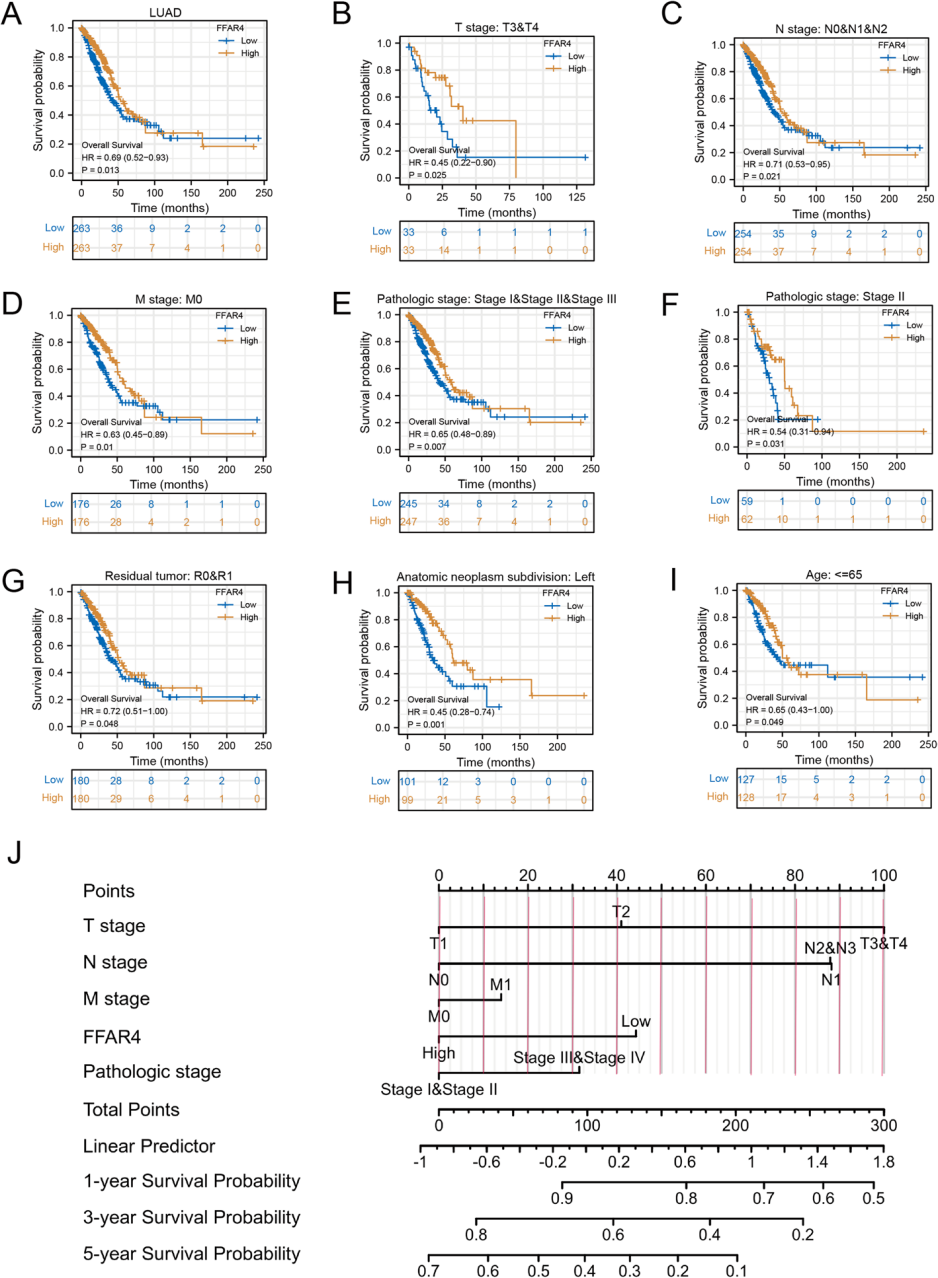
Relationship between *FFAR4* expression and patient overall survival in patients with LUAD. **A** Kaplan–Meier plot of the expression level of *FFAR4* in the overall cohort of patients with LUAD. **B**–**I** Subgroup analyses for patients with T stage: T3 and T4; N stage: N0, N1, and N2; M stage: M0; pathological stage: stage I, II, and III; pathological stage: stage II; residual tumor: R0 and R1; anatomic neoplasm subdivision: left; and age: ≤ 65 years old. **J** A nomogram that predicts the 1-, 3-, and 5-year survival rate of patients with LUAD

The patient cohort with LUAD was divided into two groups, consisting of 267 and 268 individuals, based on their *FFAR4* expression levels being either above or below the average. The distribution of their clinical characteristics can be found in Table 1. Employing the Kaplan–Meier method, we examined the correlation between FFAR4 expression levels and overall survival (OS) in patients with LUAD, revealing a significant association between lower *FFAR4* expression levels and a poorer prognosis (*P* < 0.05; Fig. 2A). Moreover, subgroup analysis confirmed the association between *FFAR4* downregulation and poorer prognosis in patients with LUAD with T stage: T3 and T4; N stage: N0, N1, and N2; M stage: M0; pathological stage: stage I, II, and III; pathological stage: stage II; residual tumor: R0 and R1; anatomical tumor partitioning: left; and age: ≤ 65 (all *P* < 0.05; Fig. 2B–I). Furthermore, subgroup analysis confirmed the diagnostic efficacy of FFAR4 in relation to various clinicopathological aspects in patients with LUAD. Furthermore, we have developed a nomogram that integrates FFAR4 expression and various clinical variables to forecast the 1-year, 3-year, and 5-year OS in patients with LUAD (Fig. 2J). These aforementioned results indicate that individuals with lower *FFAR4* expression levels in LUAD are at a significantly elevated risk of disease progression compared to those with higher *FFAR4* expression levels.

### Diagnostic value of FFAR4 in LUAD

**Fig. 3:**
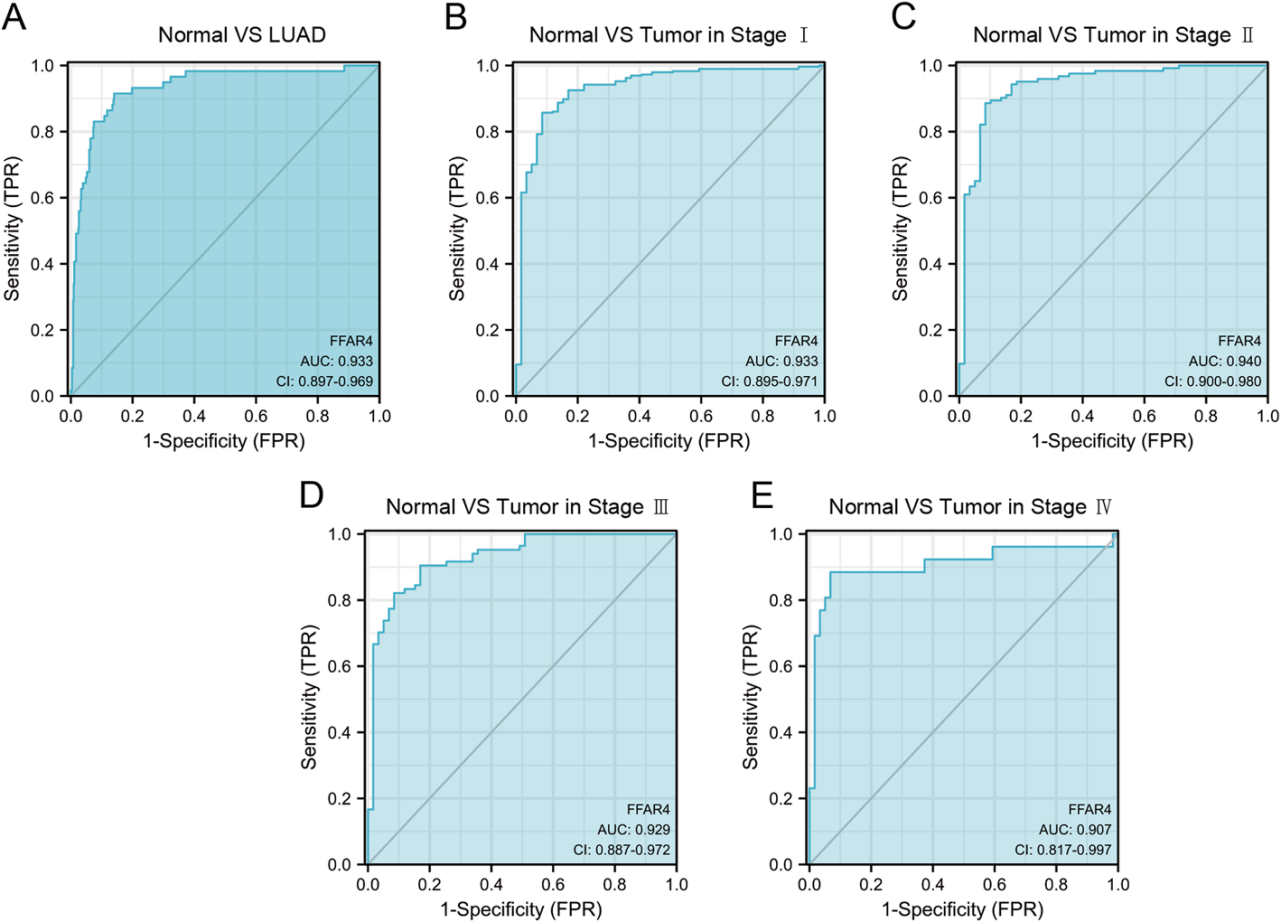
ROC analysis suggests *FFAR4* is valuable molecular biomarker for LUAD. **A** ROC curves indicated that *FFAR4* expression was a valuable indicator of the difference between tumors and normal tissues. The *X*- and *Y*-axis correspond to rates of true- and false-positive results, respectively. **B**–**E** Subgroup analyses for stage I, II, III, and IV LUAD tumors

Given the inadequate survival rates and elevated mortality associated with LUAD, the identification of specific biomarkers for the diagnosis and treatment of patients with LUAD is an urgent imperative. The diagnostic value of *FFAR4* expression as a biomarker for LUAD was investigated using the ROC curve. Figure 3A demonstrates an AUC score of 0.933, confirming its efficacy in accurately distinguishing LUAD from normal tissue. *FFAR4* was also depicted as a biomarker capable of differentiating tumor stage, with AUC values of 0.933, 0.940, 0.929, and 0.907 for stage I, II, III, and IV LUAD tumors, respectively (Fig. 3B–E). These findings suggest that FFAR4 may serve as a valuable biomarker in the diagnosis of LUAD.

### FFAR4 activation inhibits proliferation and induces cell cycle arrest in LUAD

**Fig. 4:**
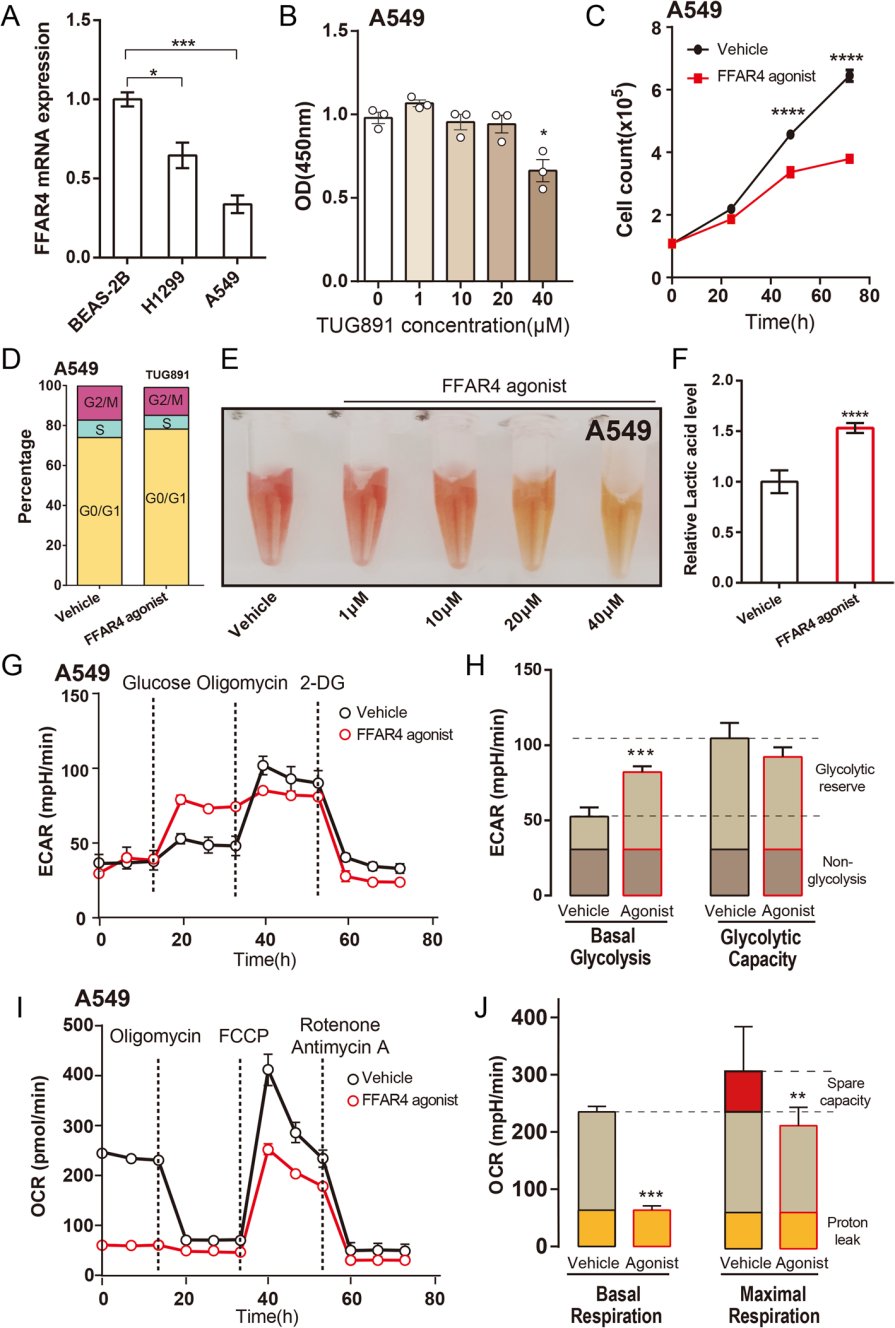
FFAR4 activation inhibits lung adenocarcinoma cell proliferation and induces metabolic shift in A549 cells. **A** Comparison of FFAR4 expression in three cell lines. **B** The effect of different concentration of TUG891 on proliferation of A549 cells. **C** Effects of 40 μM TUG891 treatment on A549 cell proliferation at 24, 48, and 72 h. **D** Effects of FFAR4 activation on cell cycle transition in A549 cells. **E** Color changes in A549 medium treated with different concentrations of TUG891. **F** Changes in the lactate content of the medium after treatment with 40 μM TUG891. **G**–**J** Effect of 40 μM TUG891 agonist on oxygen consumption rate (OCR) and extracellular acidification rate (ECAR) in A549 cells. Data are expressed as the mean ± SEM. *P* < 0.05 was considered statistically significant using two-way ANOVA; NS, not significant

To further elucidate the role of FFAR4 in LUAD, a series of in vitro cellular experiments were conducted. *FFAR4* expression levels were downregulated in lung adenocarcinoma cell lines H1299 and A549 compared with normal lung epithelial cell line BEAS-2B. A549 cells, which exhibited the lowest *FFAR4* expression, were utilized to investigate the impact of FFAR4 on LUAD function (Fig. 4A). We first performed FFAR4 overexpression in two cell lines and showed that FFAR4 overexpression had a slight inhibitory effect on A549 cell proliferation (Additional file 2: Fig. S2A–D).

A549 cells were subjected to treatment with varying concentrations of TUG891, a widely utilized agonist targeting FFAR4, for a duration of 72 h (Fig. 4B). Subsequent to TUG891 treatment, cell proliferation was observed to be significantly impeded in comparison with the vehicle group (Fig. 4C). Furthermore, flow cytometry analysis was employed to assess the impact of TUG891 treatment on the cell cycle. Notably, TUG891 treatment elicited noteworthy alterations in the cell cycle, as evidenced by an increase in the number of cells in the G0/G1 phase and a decrease in the number of cells in the S and G2/M phases (Fig. 4D). A similar phenomenon was found in H1299 cells (Additional file 2: Fig. S2E–G). In summary, activation of FFAR4 exhibited a substantial inhibitory effect on the proliferation of lung adenocarcinoma cells and induced cell cycle arrest.

### FFAR4 activation severely diminished OXPHOS capacity and increased cellular glycolysis in A549 cells

Although FFAR4 activation significantly inhibited the proliferation of lung adenocarcinoma cells, the underlying mechanism is unclear. Notably, the TUG891-treated group exhibited a prominent color change in the cell culture medium during the cell culture process, prompting further investigation (Fig. 4E; Additional file 2: Fig. S2H). We hypothesize that FFAR4 activation may be linked to the reprogramming of cancer metabolism, particularly the enhancement of glycolysis resulting in increased lactic acid production. The medium of the FFAR4 agonist group showed a significant increase in lactic acid levels compared with the control group (Fig. 4F; Additional file 2: Fig. S2I), which was a direct manifestation of the enhanced glycolysis. The activation of FFAR4 leading to enhanced glycolysis suggests an increased energy demand in tumor cells. The inhibitory effect of TUG891 on A549 cells may be attributed to an energy gap resulting from excessive consumption of medium nutrients. However, the addition of glucose, pyruvate, glutamine (Gln), palmitic acid (PA), oleic acid (OA), or fresh media did not alleviate the inhibition of A549 cell proliferation by TUG891 (Additional file 3: Fig. S3A-B).

To further investigate the effect of TUG891 on energy expenditure patterns in A549 cells, the Seahorse extracellular flux analyzer was employed to measure the extracellular acidification rate (ECAR) and oxygen consumption rate (OCR) in lung adenocarcinoma cells. The findings revealed that treatment with TUG891 resulted in a facilitative effect on the basal level of glycolysis (Fig. 4G, H), as well as a noticeable suppressive effect on both the basal level and the maximal capacity associated with oxidative respiration (Fig. 4I, J) in A549 cells. This suggests that the growth arrest and reduced proliferation caused by FFAR4 activation may be a result of alterations in glycolysis and OXPHOS metabolism.

### FFAR4 activation induces metabolic shifts via mitochondrial complex assembly blockage

**Fig. 5:**
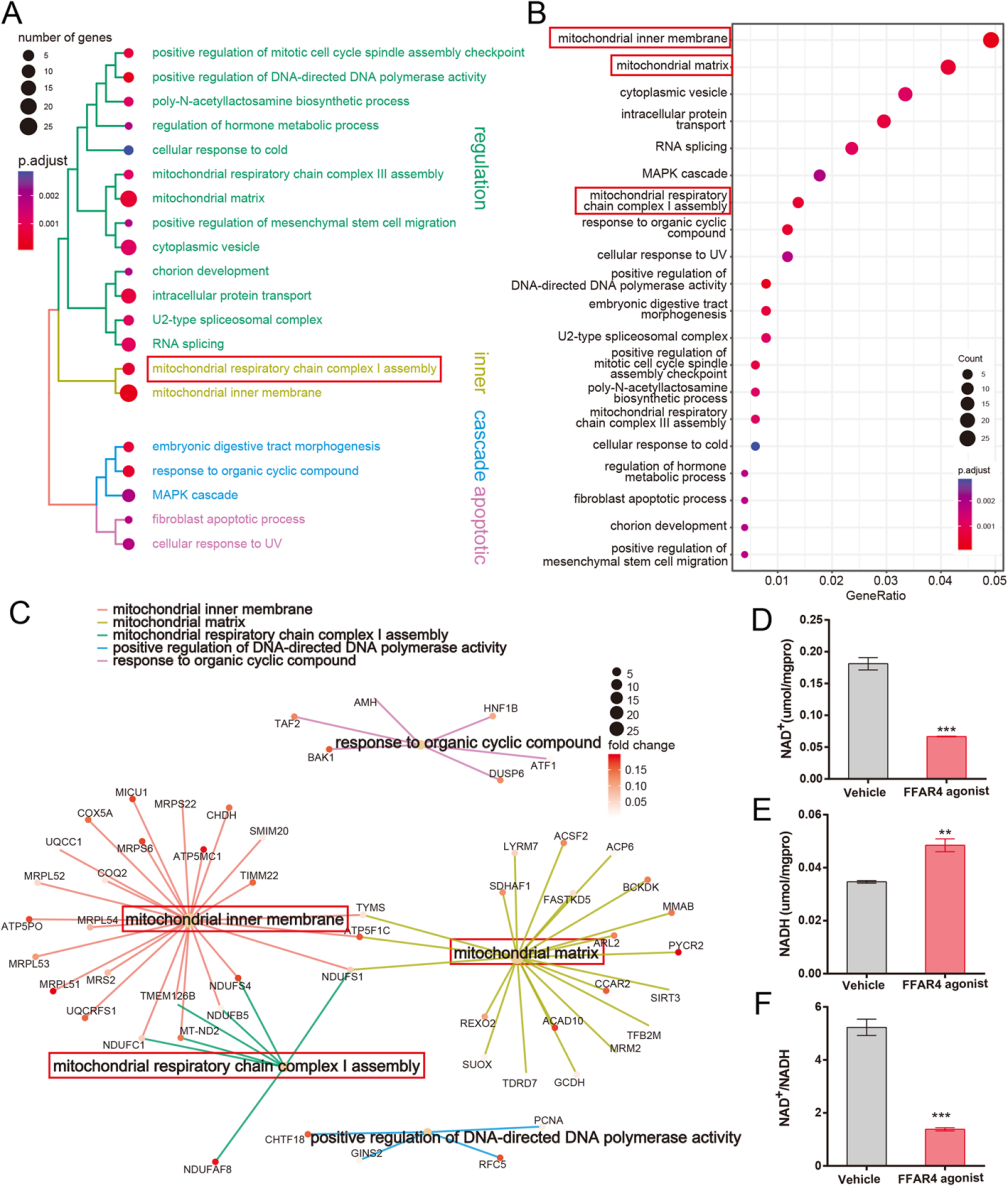
FFAR4 activation leads to reduction of NAD^+^/NADH ratio by affecting mitochondrial complex I assembly. **A**–**C** Tree plot, dot plot, and cnet plot of the results of ORA (GO analysis) of downregulated genes after FFAR4 activation. The size of the dots indicates the number of genes in a particular pathway and the color indicates the correlation with the adjusted *P* value. **D**–**F** NADH and NAD^+^ levels and the NAD^+^/NADH ratio were measured from A549 cells between the vehicle group and FFAR4 activator group

To further elucidate the potential mechanisms underlying the metabolic alterations resulting from FFAR4 activation, a transcriptomic analysis was conducted. The findings of the transcriptomic study revealed a strong correlation between the downregulated genes following FFAR4 activation and mitochondrial gene expression, as well as alterations in mitochondrial membrane components (Fig. 5A, B). Additionally, pathway analysis demonstrated that FFAR4 activation directly impacts the initial step of mitochondrial oxidative phosphorylation (OXPHOS), specifically the assembly of mitochondrial complex I (*NDUFAF8*, *NDUFC1*, *MT-ND2*, *TMEM126B*, *NDUFB5*, *NDUFS4*, and *NDUFS1*; Fig. 5C). Impaired assembly of mitochondrial complex I will directly impact the transfer of high-energy electrons in NADH within the mitochondria, thereby affecting electron transfer downstream of the electron transport chain (ETC). Impaired assembly of mitochondrial complex I will directly impact the transfer of high-energy electrons in NADH within the mitochondria, thereby affecting electron transfer downstream of the electron transport chain (ETC; Fig. 5D–F). Furthermore, the analysis of human transcriptomic data from TCGA through pathway enrichment unveiled that the cluster of genes linked to FFAR4 exerts a repressive influence on various mitochondrial respiratory chain processes, such as NADH dehydrogenase complex assembly (Additional file 4: Fig. S4A–D). This finding further substantiates the strong association between FFAR4 and mitochondrial energy metabolism processes in lung adenocarcinoma. Importantly, our data does not demonstrate any augmentation of crucial genes involved in glycolytic metabolism, leading us to propose that the activation of FFAR4 triggers a feedback effect resulting in the enhancement of glycolysis, which originates from a cellular energy gap caused by disruption of the mitochondrial OXPHOS process.

### Malate–aspartate shuttle process may participate in FFAR4 activation-induced reduction of NAD^+^/ NADH transition

**Fig. 6:**
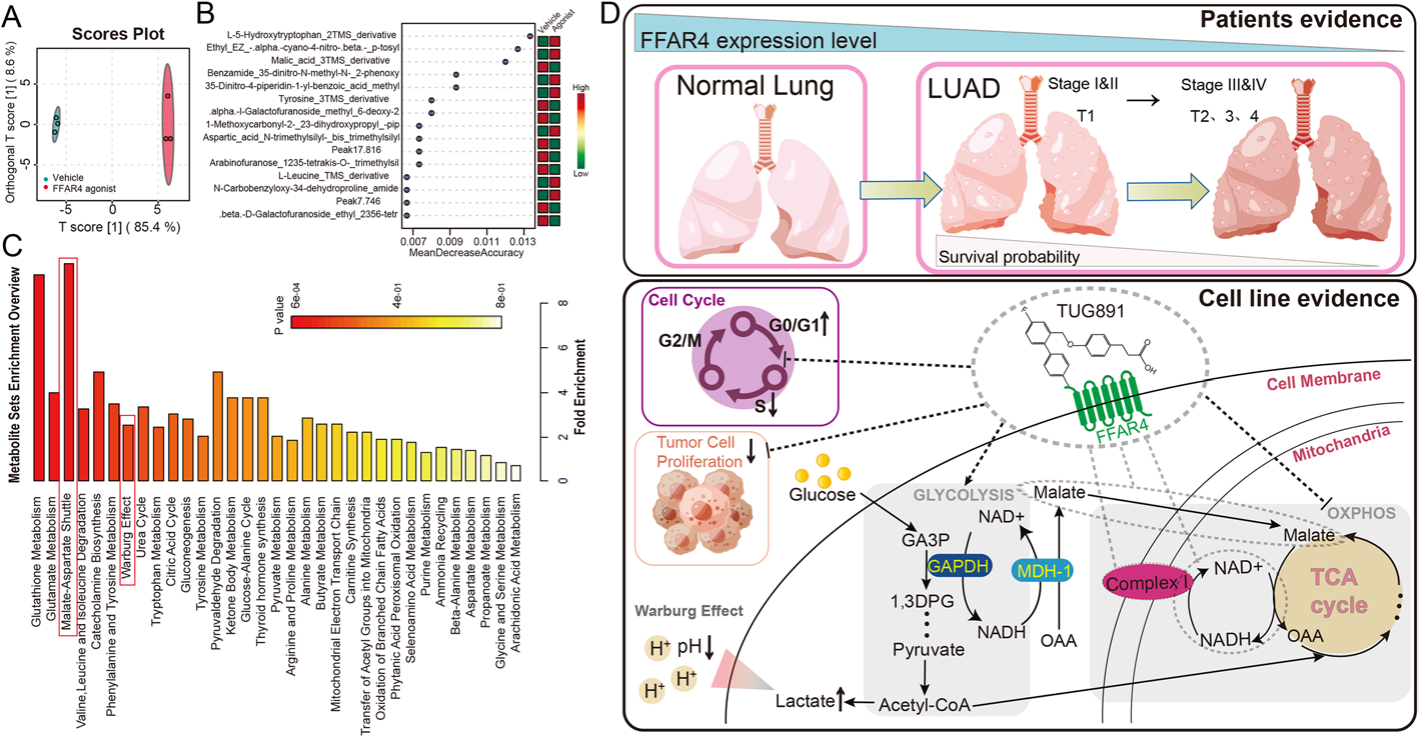
FFAR4 activation enhances malate–aspartate shuttling in A549 cells. **A** Overall differences between groups were demonstrated by the using orthogonal partial least-squares discriminant analysis (OPLS-DA). **B** A random forest approach was used to select the features with the highest contribution to the accuracy of the sample grouping predictions. **C** Based on the significantly different metabolites, ORA was used to find KEGG pathways that were significantly enriched in metabolites. **D** Conceptual summary

Additionally, untargeted metabolomic analyses were conducted to elucidate the metabolic differences between the groups treated with the FFAR4 agonist and the control group. The application of orthogonal partial least-squares discriminant analysis (OPLS-DA) yielded score plots that effectively distinguished the FFAR4 agonist treated group from the vehicle group (Fig. 6A). To identify the metabolites that significantly influenced the accuracy of sample grouping predictions, a random forest approach was employed, as depicted in Fig. 6B. Furthermore, the analysis of KEGG pathways revealed that the malate–aspartate shuttle process exhibited the highest enrichment following FFAR4 activation (Fig. 6C). Upon entry into the mitochondria from the cytoplasm, malic acid actively engages in the mitochondrial tricarboxylic acid (TCA) cycle, contributing its hydrogen ions to NAD^+^ and thereby converting it to NADH. This process intensifies the existing imbalance of the NAD^+^/NADH ratio within the mitochondria. In light of our findings, it can be inferred that the reduced mitochondrial NAD^+^/NADH ratio resulting from FFAR4 activation is attributable to two factors: first, the impediment of mitochondrial complex I assembly, and second, the expedited conversion of NAD^+^ to NADH facilitated by the malate–aspartate shuttle process.

## Discussion

The significance of LCFA, a crucial constituent of the human diet, has been extensively examined in the progression of lung adenocarcinoma25. FFAR4, a distinct receptor for LCFA, potentially plays a role in LUAD by connecting genetic and environmental factors28. Through our comprehensive bioinformatics analysis of *FFAR4*, we have observed a substantial reduction in *FFAR4* expression in LUAD, which correlates with unfavorable patient prognosis and diminished survival rates. Our experimental findings demonstrate that the activation of FFAR4 in lung adenocarcinoma cells leads to a noteworthy decline in the NAD^+^/NADH ratio and leads to dysfunction of OXPHOS, disrupting cellular energy balance and slowing down the proliferation of lung adenocarcinoma cells. These results collectively illustrate that FFAR4 activation reprograms virtually mitochondrial metabolism within lung adenocarcinoma cells (Fig. 6D).

Metabolic reprogramming, referred to as certain metabolic transformations, is frequently observed in tumor cells and is regarded as indicative of cancer [36, 37]. The Warburg effect, a prevalent metabolic phenotype in LUAD, has been identified [38, 39], and the targeting of the mitochondrial OXPHOS metabolic pathway has demonstrated efficacy in impeding LUAD progression [40]. Metformin, recognized as a mitochondrial OXPHOS inhibitor, has been reported to effectively decrease LUAD morbidity and mortality in clinical investigations [41–43]. The concurrent administration of metformin and a glycolysis inhibitor, such as WZB117, has been found to result in a notable reduction in intracellular ATP synthesis and subsequent demise of lung cancer cells [44]. Our findings indicate that the FFAR4 agonist exhibits comparable effects to metformin. The primary mechanism of action of the FFAR4 agonist involves targeting the assembly of mitochondrial complex I, thereby influencing the electron transport process within the mitochondria. Consequently, this disruption impedes the oxidative phosphorylation process and hampers the provision of sufficient energy to A549 cells. Furthermore, the findings indicate that the activation of FFAR4 agonist had a marginal impact on the glycolytic capacity of lung adenocarcinoma cells, potentially due to insufficient energy provision. Consequently, it is hypothesized that the concurrent administration of FFAR4 agonist and glycolysis inhibitors could serve as a viable therapeutic approach for lung adenocarcinoma.

According to reports, LUAD is strongly linked to metabolic disorders and hyperinsulinemia [45]. Various drugs have been found to mitigate the stimulatory effects of hyperinsulinemia on lung cancer growth by enhancing insulin sensitivity and reducing circulating insulin levels [46, 47]. Interestingly, TUG891 has a comparable effect in enhancing insulin sensitivity and reducing circulating insulin levels [48, 49]. Based on that evidence, it is plausible that FFAR4 agonists could play a dual role in tumor and metabolism regulation, thereby holding significant promise in the field of oncology drug development. Further investigation is warranted to comprehensively explore the molecular mechanisms through which FFAR4 modulates the metabolic pathway in LUAD.

Multiple studies have demonstrated the presence of various *FFAR4* mutations within populations [50, 51], and it is crucial to acknowledge that FFAR4 ligands can exert distinct effects on lung adenocarcinoma in these populations. The significance of this should not be disregarded, as it has implications for disease progression and treatment. Last but not least, FFAR4 may play different roles in different tumor types. FFAR4 signalling has antitumour effects in prostate and colorectal adenocarcinomas [34, 52], while it has been reported to be a procarcinogenic receptor in the development of breast cancer [53]. How to precisely target and regulate FFAR4 in tissues and organs will also be an important research direction. Consequently, the exploration of FFAR4 agonists and targeted delivery techniques holds considerable potential for the management of LUAD. Our research endeavors may stimulate interest in the domains of GPCR signaling, FFAR drug development, and the diagnosis and treatment of LUAD.

### Supplementary Information

**Additional file 1: Figure S1.** Aberrant expression of *FFAR4* is correlated with clinicopathologic findings in LUAD patients. Figures (A-G) illustrate the relationship between the expression of *FFAR4* and Pathologic stage, T Stage, Primary therapy outcome, number_pack_years_smoked and Smoker, DSS event, PFI event among LUAD patients in TCGA database. Data are expressed as the mean ± standard error of the mean. *P* < 0.05 was considered statistically significant using two-way ANOVA; NS, not significant.

**Additional file 2: Figure S2.** *FFAR4* overexpression and FFAR4 activation inhibit lung adenocarcinoma cell proliferation. (A) Validation of FFAR4 overexpression in A549 and H1299. (B, C) Effects of FFAR4 overexpression on cell proliferation in A549 and H1299 cells at 24, 48 and 72 h. (D) Color changes of A549 and H1299 media after FFAR4 overexpression treatment. (E) The effect of different concentration of TUG891 on proliferation of H1299 cells. (F) Effects of 40 μM TUG891 treatment on H1299 cell proliferation at 24, 48,72 h. (G) Effects of FFAR4 activation on cell cycle transition in H1299 cells. (H) Colour changes in H1299 medium treated with different concentrations of TUG891. (I) Changes in the lactate content of the medium after treatment with 40 μM TUG891.

**Additional file 3: Figure S3.** Effect of nutrient supplementation on the proliferation inhibitory effect of TUG891. (A) Effect of glucose, pyruvate, glutamine (Gln), palmitic acid (PA), oleic acid (OA) supplementation on the antitumor effect of TUG891. (B) The effect of maintaining the pH balance of the medium by using NaHCO_3_ or replacement of the medium on the antitumor effect of TUG891. Data are expressed as the mean ± standard error of the mean. *P* < 0.05 was considered statistically significant using two-way ANOVA; NS, not significant.

**Additional file 4: Figure S4.** GSEA and Gene Ontology (GO) enrichment analysis of *FFAR4*-related genes in LUAD. (A) GO Analysis (Cellular component) and GSEA of *FFAR4*-related genes in LUAD; (B) GO Analysis (Biological process) and GSEA of *FFAR4*-related genes in LUAD.

## Data Availability

The datasets used and/or analyzed during the current study are available from the corresponding author on reasonable request.

## Abbreviations

LUAD: Lung adenocarcinoma
FFARs: Free fatty acid receptors
FFAR4: Free fatty acid receptor 4
OS: Overall patient survival
ROC: Receiver operating characteristic curve
OCR: Oxygen consumption rate
ECAR: Extracellular acidification rate
OXPHOS: Oxidative phosphorylation
GPCRs: G protein-couple protein-coupled receptors
LCFAs: Long-chain fatty acids
TPM: Transcripts per million
qPCR: Quantitative real-time polymerase chain reaction
GO: Gene Ontology
GSEA: Gene set enrichment analysis
LC: Lung cancer
CRC: Colorectal cancer
SCLC: Small cell lung cancer
NSCLC: Non-small cell lung cancer
AUC: Area under the curve
Gln: Glutamine
PA: Palmitic acid
OA: Oleic acid
ETC: Electron transport chain
OPLS-DA: Orthogonal partial least-squares discriminant analysis
TCA: Tricarboxylic acid
