# Supplementary figures and images for: FFAR4 activation inhibits lung adenocarcinoma via blocking respiratory chain complex assembly associated mitochondrial metabolism
Source: Cell Mol Biol Lett. 2024 Jan 19;29:17. doi: 10.1186/s11658-024-00535-3 (PMC10799372; doi:10.1186/s11658-024-00535-3)

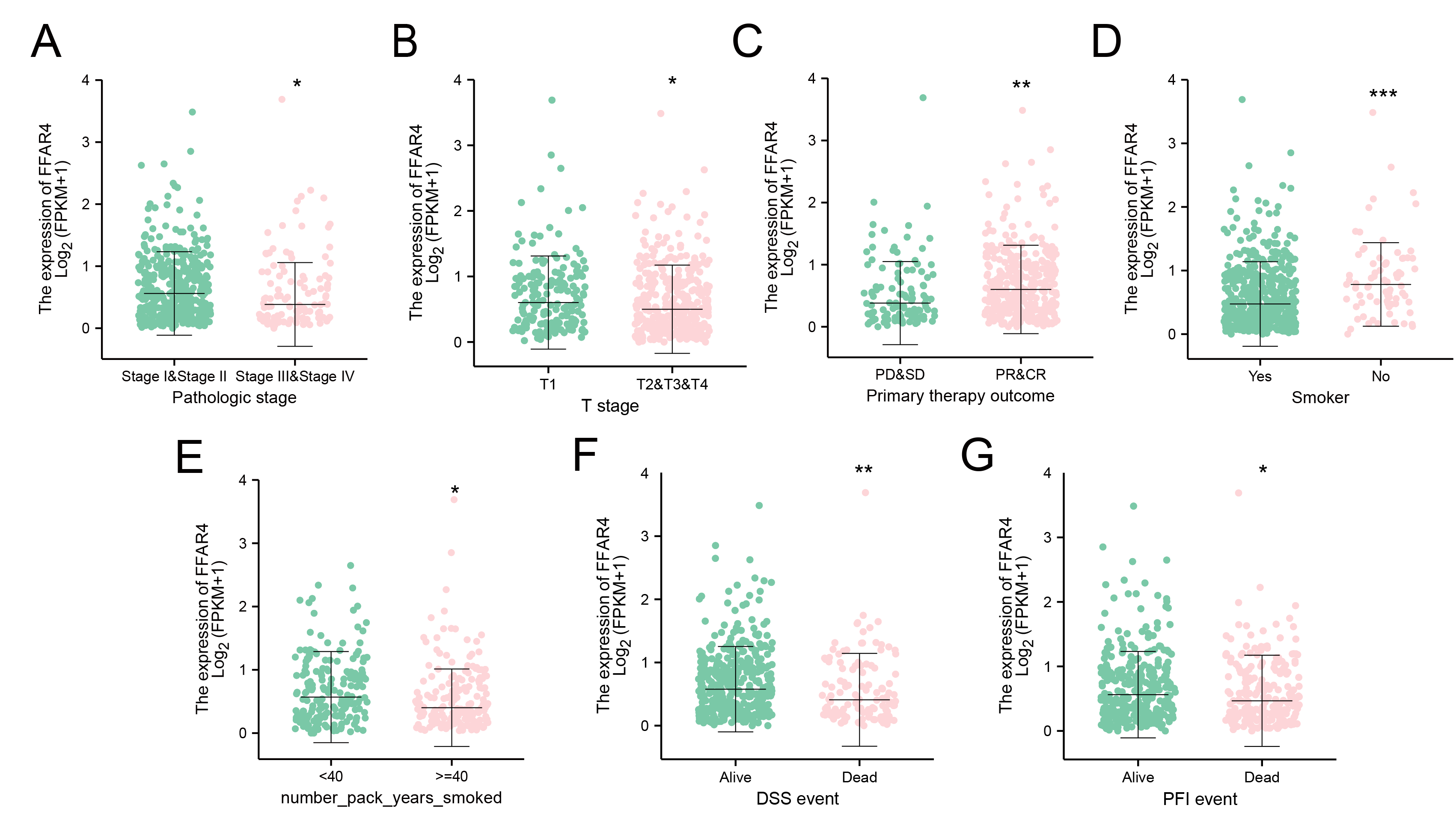

Supplement: Supplementary file 1 — Additional file 1: Figure S1. Aberrant expression of FFAR4 is correlated with clinicopathologic findings in LUAD patients. Figures (A-G) illustrate the relationship between the expression of FFAR4 and Pathologic stage, T Stage, Primary therapy outcome, number_pack_years_smoked and Smoker, DSS event, PFI event among LUAD patients in TCGA database. Data are expressed as the mean ± standard error of the mean. P < 0.05 was considered statistically significant using two-way ANOVA; NS, not significant. [file 11658_2024_535_MOESM1_ESM.tif]

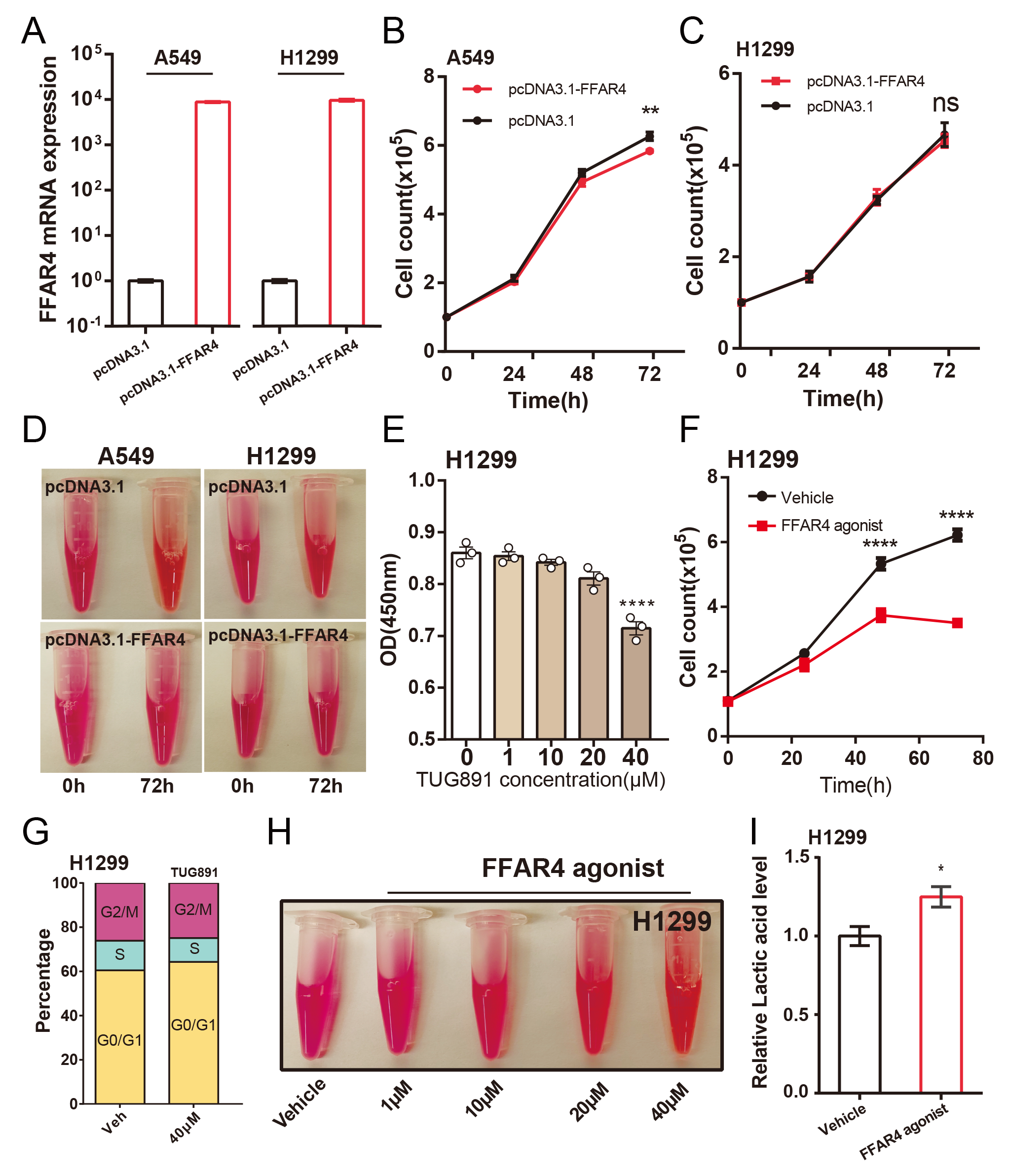

Supplement: Supplementary file 2 — Additional file 2: Figure S2. FFAR4 overexpression and FFAR4 activation inhibit lung adenocarcinoma cell proliferation. (A) Validation of FFAR4 overexpression in A549 and H1299. (B, C) Effects of FFAR4 overexpression on cell proliferation in A549 and H1299 cells at 24, 48 and 72 h. (D) Color changes of A549 and H1299 media after FFAR4 overexpression treatment. (E) The effect of different concentration of TUG891 on proliferation of H1299 cells. (F) Effects of 40 μM TUG891 treatment on H1299 cell proliferation at 24, 48,72 h. (G) Effects of FFAR4 activation on cell cycle transition in H1299 cells. (H) Colour changes in H1299 medium treated with different concentrations of TUG891. (I) Changes in the lactate content of the medium after treatment with 40 μM TUG891. [file 11658_2024_535_MOESM2_ESM.tif]

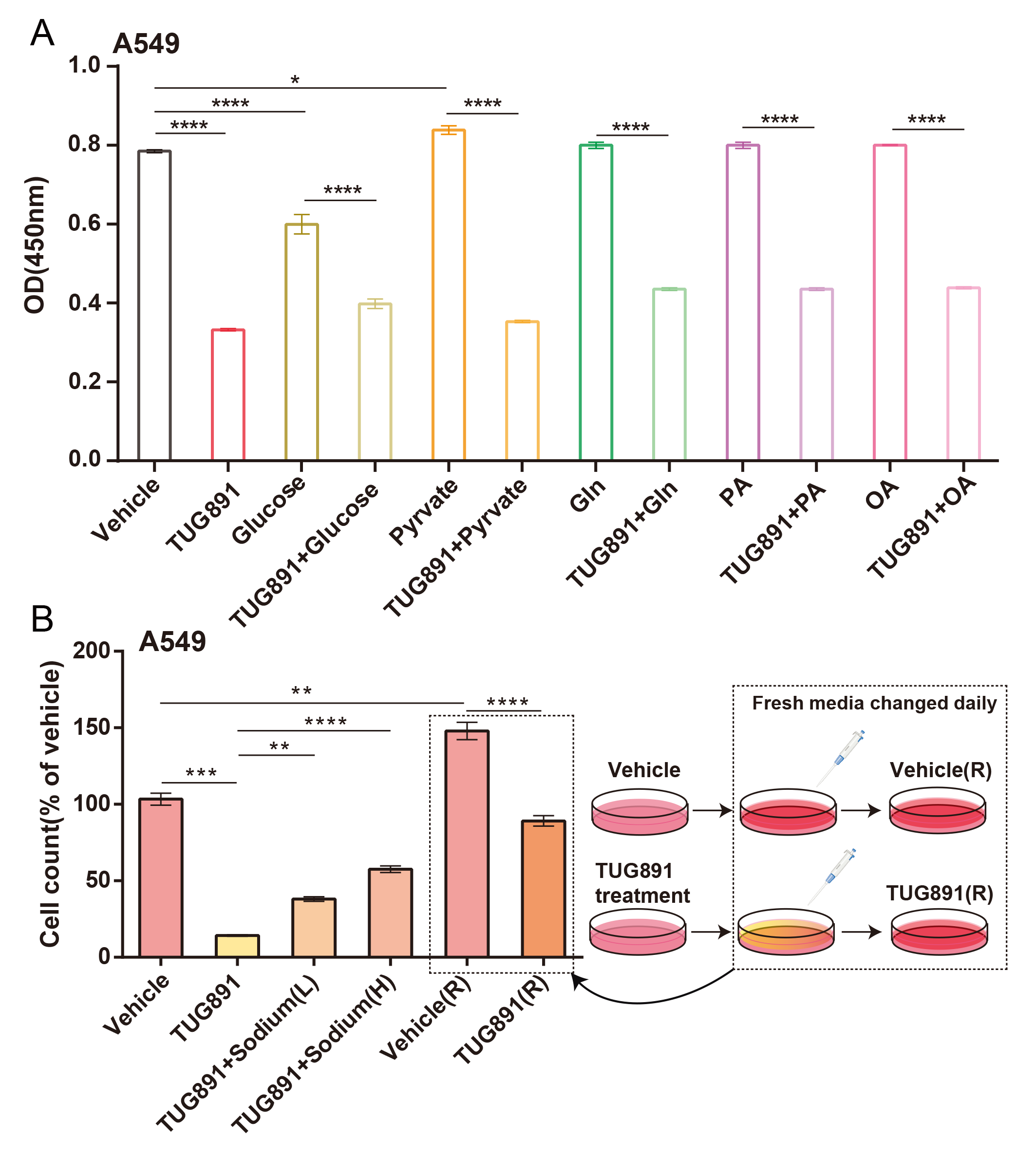

Supplement: Supplementary file 3 — Additional file 3: Figure S3. Effect of nutrient supplementation on the proliferation inhibitory effect of TUG891. (A) Effect of glucose, pyruvate, glutamine (Gln), palmitic acid (PA), oleic acid (OA) supplementation on the antitumor effect of TUG891. (B) The effect of maintaining the pH balance of the medium by using NaHCO3 or replacement of the medium on the antitumor effect of TUG891. Data are expressed as the mean ± standard error of the mean. P < 0.05 was considered statistically significant using two-way ANOVA; NS, not significant. [file 11658_2024_535_MOESM3_ESM.tif]

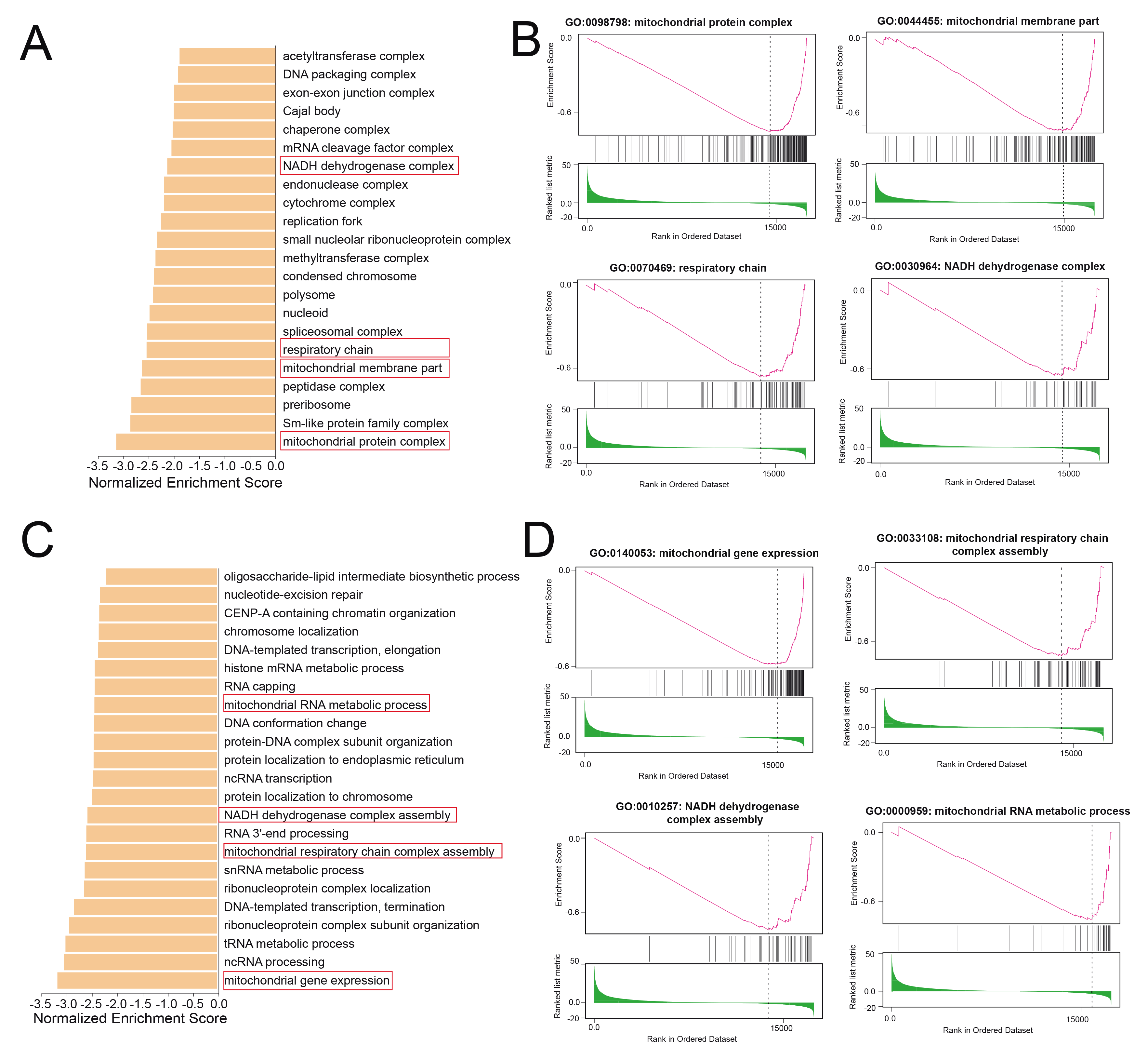

Supplement: Supplementary file 4 — Additional file 4: Figure S4. GSEA and Gene Ontology (GO) enrichment analysis of FFAR4-related genes in LUAD. (A) GO Analysis (Cellular component) and GSEA of FFAR4-related genes in LUAD; (B) GO Analysis (Biological process) and GSEA of FFAR4-related genes in LUAD. [file 11658_2024_535_MOESM4_ESM.tif]
